# Clinician perspectives about delivering a blended care treatment model for adolescent depression: a qualitative study

**DOI:** 10.1186/s13034-025-00989-z

**Published:** 2025-11-21

**Authors:** Alexandra J. South, Adam Theobald, Brittany Corkish, Sophie Li, Melinda Achilles, Mirjana Subotic-Kerry, Bridianne O’Dea, Aliza Werner-Seidler

**Affiliations:** 1 Black Dog Institute, University of New South Wales, Sydney, NSW Australia; 2 School of Psychology, University of New South Wales, Sydney, NSW Australia; 3 Flinders Institute for Mental Health and Wellbeing, College of Education, Psychology and Social Work, Flinders University, Adelaide, Australia

**Keywords:** Cognitive behavioural therapy, Blended care, Depression, Digital intervention

## Abstract

**Background:**

Availability of psychological services for adolescents cannot keep up with demand. Integrating digital interventions with face-to-face care into a blended model, an approach to treatment that incorporates the benefits of both digital and face-to-face therapy, may help address some access barriers by aiming to optimise treatment efficiency and effectiveness. This study explored mental health professionals' perspectives on incorporating a CBT-based app (ClearlyMe®) into their clinical practice with adolescents experiencing depression.

**Methods:**

Semi-structured focus groups and interviews were conducted with 37 mental health professionals (psychologists, counsellors, social workers) working with adolescents in schools or private practices. ClearlyMe® is a self-directed, CBT-based smartphone app designed to address depressive symptoms among adolescents. Participants trialed the ClearlyMe® app for 10 min prior to focus groups and interviews. The discussions explored how mental health professionals would prefer to integrate ClearlyMe® into their face-to-face therapy when working with adolescents with depression, as well as the training and support requirements for doing so. Data was analysed using a deductive thematic analysis approach.

**Results:**

Five key themes were identified: (1) Optimal ways to integrate the app with therapy; (2) Suitability of clients for blended care; (3) Facilitators and barriers to adopting blended care; (4) Data and privacy issues; and (5) Training and support requirements for those delivering care.

**Conclusions:**

Clinicians supported ClearlyMe® in a blended care format if it were to complement their practice without adding workload and was supported by flexible training. They emphasized caution with data sharing and noted the model would be best suited to motivated clients, with at-risk clients likely needing more support. Future work will develop a blended care framework and training resources.

**Supplementary Information:**

The online version contains supplementary material available at 10.1186/s13034-025-00989-z.

## Introduction

Approximately one in four adolescents experience elevated symptoms of depression at any given time [19]. Rates of adolescent mental health issues are increasing, representing the leading health burden among young people [27] with lasting impacts on developmental trajectories and functioning [17]. However, relatively few adolescents access timely professional help, with young people globally experiencing long wait times in increasingly overburdened health systems [22], with a mean wait time of over 3 months to access mental health services reported in an Australian adolescent sample [23]. Furthermore, depression carries a substantial risk of recurrence and chronicity and is a significant risk factor for youth suicide [18]. These challenges highlight the need for effective and accessible treatment options.

Cognitive behavioural therapy (CBT), traditionally delivered face-to-face by trained mental health professionals, is considered one of the gold-standard treatment for depression [6]. There is strong evidence that self-guided, digitally delivered CBT interventions are also effective in reducing symptoms of depression in adolescents [4, 7]. Digitally delivered CBT may therefore offer a viable alternative when in-person therapy is limited by barriers such as cost, time, and transport availability [10]. However, face-to-face therapy has several advantages over digital-only treatments, including the benefits of a therapeutic relationship, increased engagement with the therapeutic material, and the ability to identify and respond to risk [1]. Blended care is an emerging treatment approach whereby digital interventions are integrated with face-to-face therapy to enhance engagement and treatment outcomes [20]. While definitions of what is truly ‘blended’ care vary, in a common conceptualization, the digital component of blended treatments (generally accessible via computers or mobile devices), provides self-directed psychoeducation modules and exercises, with therapists offering guidance and feedback [8]. Face-to-face sessions focus on relationship building and preparing individuals/clients for the digital component [11, 20, 24]. Blended care approaches may reduce the number of face-to-face sessions needed for therapeutic benefit, offering a more time- and cost-efficient option than traditional face-to-face therapy. Research investigating blended care models in adolescents has revealed that although young people generally indicate a preference for face-to-face treatment, those who have received blended care report positive experiences [21]. Blended care models are commonly used in adult mental health care [8, 9, 25], while options for adolescents remain limited [20]. Importantly, it is unclear whether blended care models require specific adaptations to meet the needs of adolescents.

Researchers at the Black Dog Institute developed ClearlyMe®, a CBT smartphone application targeting depressive symptoms among adolescents [14]. In a randomized controlled trial, participants aged 12–17 years allocated to use ClearlyMe® demonstrated greater reductions in depressive symptoms relative to an active control group [16]. The purpose of the current study is to understand mental health professionals’ perspectives and preferences regarding the development of a blended care treatment model that integrates a digital CBT evidence-based intervention (ClearlyMe®) with face-to-face therapy to treat depression in adolescents.

Systematic reviews show that blended interventions can effectively reduce depression in adults [8]. However, much less has been done in young people, the models tested vary in terms of level of clinician support, and implementation models used [21]. Addressing this gap is critical, as clinicians are central to the successful uptake and implementation of blended interventions, and limited perceived value or applicability may impede uptake or engagement with the treatment model [12].

This study is guided by two key research questions: How can digital interventions be integrated into face to face therapy for young people? Second, what are the information, data, and training requirements for mental health professionals in delivering a blended care model?

## Methods

### Participants and sampling

The participants were mental health professionals who have self-identified as having experience delivering psychological interventions to adolescents. They were recruited via an invitation email sent to members of an existing Black Dog Institute mailing list (mental health professionals who had ‘opted-in’ to receive research communications from the Black Dog Institute, N = 159) as well as through other professional networks. Recipients were encouraged to forward the invitation to their professional networks using a snowball sampling approach. The participants were reimbursed with digital gift cards to the value of $150 per hour of participation in recognition of their time and expertise.

The inclusion criteria included being a mental health professional (e.g., counsellor, psychologist), fluent in English, living in Australia, and currently providing mental healthcare to adolescents with depression in a private practice, school, or similar setting. Participants also needed access to a device with a camera and microphone for teleconferencing, access to a smartphone capable of installing and running the ClearlyMe® app, and willingness to provide informed consent for audio recording. There were no specific exclusion criteria. This project was reviewed and approved by the University of New South Wales Human Research Advisory Panel (iRECS6823).

#### Participant demographics

Fifty-five individuals responded to the initial invitation email and survey. The final sample consisted of 37 participants who were available to attend an online focus group or interview during the recruitment period. The participants were predominantly female (86%, 11% male, and 3% nonbinary) and had an average of 7.2 years of experience working with adolescents. They worked primarily in school settings (49%), private practice (46%), or other similar settings (5%). Most participants were psychologists (75%), followed by counsellors (14%) and social workers (11%). While only a minority of clinicians reported regularly integrating or referring clients to digital interventions (13.51% always, 10.81% most of the time), the vast majority (83.78%) indicated they were very open to doing so. Data was collected via seven focus groups (2–5 participants each) and 14 individual interviews.

### Data collection

#### Interview guide/focus group questions

The interviews and groups followed a semi-structured approach that covered the key research questions (*see Supplementary Material*). The questions focused on perspectives on treatment structure; training and support needs; strategies for integrating the ClearlyMe® digital intervention into existing face-to-face therapy; criteria for determining client suitability for blended care; the appropriate balance between a prescriptive versus flexible treatment model; and the clinical utility of app-derived data.

#### Data collection procedure

The participant information statement and consent form were presented via Qualtrics. After providing consent, participants completed a brief survey assessing demographics, professional qualifications and experience, current role, and availability for a focus group or interview. The participants were contacted directly to schedule a focus group or interview. Before attending, they were asked to download the ClearlyMe® app to their personal device. The app is publicly available in Australia via Apple and Google Play stores at no cost. All focus groups and interviews were conducted via Zoom by a member of the research team (AS, AT and/or AWS). With participant consent, all sessions were audio recorded for later transcription.

Prior to the interviews, clinicians were instructed to explore the ClearlyMe® app which was assigned as prework. At the beginning of the focus group or interview, the interviewer provided a brief introduction to the major app features, navigation, and definition of blended care. Given that we expected variability in the extent to which clinicians would have engaged with the app prior to the session as instructed, participants were then given 10 min to spend with the app with the app. While some clinicians had already used or explored it in detail, the brief trial allowed all participants to become familiar with its functionality prior to the discussion. Next, the interviewer commenced the discussion in line with the interview guide. Upon completion of the interview or focus group, the participants were emailed a gift card.

### Data preparation and analysis

Audio recordings were transcribed by an Australian-based professional transcription company, Digital and Audio Transcription Services (DAATS). Personal and school identifiers were removed during transcription. The dataset consisted of 21 transcripts: seven from focus groups, and 14 from individual interviews. A random subset of transcripts was reviewed by AT to ensure accuracy.

#### Research reflexivity

An important part of the qualitative analysis process is reflexivity and awareness of one’s own perspectives [2]. Researchers involved in the project remained mindful of their own perspectives when developing response categories and interpreting and coding the data. The coding team consisted of three females and one male with diverse research and clinical backgrounds. The primary interviewer and coder (AS) is a PhD student in medicine with experience in personality and mental health research. The secondary interviewer and coder (AT) has a master’s degree in clinical psychology and has experience with digital and adolescent mental health research. The third coder (BC) is a clinical psychologist and research officer. The principal investigator (AWS) is a senior researcher and clinical psychologist specializing in adolescent mental health.

#### Thematic analysis

A deductive thematic analytical approach was used to identify information from the transcripts that directly mapped onto the two overarching research questions. This approach was informed by the six-phase approach to thematic analysis outlined by Clarke and Braun [2, 3] [5]. While Clarke and Braun’s method is often associated with inductive approaches, its systematic and flexible nature allows for adaptation to deductive coding, enabling the integration of a-priori concepts with emergent patterns from the data. Three coders (AS, AT, and BC) conducted the coding process via Excel. Initially, AS and AT independently reviewed five transcripts each and created a draft coding framework. They then met with AWS to discuss and review the framework. After consulting with AWS, AS and AT refined the framework, which was subsequently reviewed by AWS. AS and AT then tested the refined framework by dual coding one transcript to determine interrater alignment. A high level of agreement was established between researchers to ensure consistency in coding. Each transcript was then double coded and discussed by the coders (AS, AT, BC) to ensure alignment and resolve disagreements.

## Results

### Overview of themes

The analysis identified five key themes: (i) optimal ways to integrate the app with therapy; (ii) the suitability of clients for blended care; (iii) facilitators and barriers to adopting blended care; (iv) data and privacy issues; and (v) training and support requirements for those delivering the care. The themes are presented in Table 1 through 5, along with subthemes and illustrative quotes.

### Themes and subthemes

#### Optimal ways to integrate the app with therapy

This theme summarizes clinicians’ perspectives on possible approaches to incorporating a digital intervention within routine care. Clinicians discussed how they would introduce the app to ensure client engagement and buy-in, as well as when and where the digital intervention might be most effectively used. Given the highly diverse needs of clients, flexibility was emphasized by participants as a key requirement. Within this theme, five subthemes were identified, as outlined below.

##### Introduction to the digital intervention

The way the digital intervention is introduced to clients was seen by all clinicians as an important step that was best managed through professional discretion in timing and tailoring. Clinicians anticipated that introduction of the digital intervention would likely occur after rapport-building and psychoeducation, alongside their assessment of a client’s motivation to engage in a blended care model. Some suggested they might introduce the app at the end of a first session, while others envisioned doing so at the beginning of a second session. All would demonstrate the use of the app in person (the level of detail depending on individual client needs). The ‘mood tracking’ feature was popular amongst the clinicians, noted as a simple way for clients to start engaging with the intervention, which was synonymous with the approach many clinicians would take in their usual practice.

##### Typical pattern/duration of contact

Adapting a digital intervention to the typical cadence of sessions was an important topic of discussion for all clinicians, given the heterogeneity of practice across different settings. Clinicians working in private practice consistently reported that their pattern of contact with adolescents is largely structured by *Medicare* (Australia’s public health system), which provides up to 10 subsidized psychological sessions per year under a Mental Health Care Plan (with referral from a general practitioner/physician). The private clinicians reflected that their adolescent clients typically received 6 to 8 sessions. A few were cautious about using ClearlyMe® with clients likely to require longer-term support, as they felt that these clients tended to have more complex presentations that may not be suitable for ClearlyMe®, given the target user group of the app. Most clinicians reported that they typically met with clients fortnightly to monthly, depending on need and preferences, and that ClearlyMe would be useful during these periods when they are not meeting with their client.

All clinicians working in schools reported highly varied patterns of contact with students, both within and between schools. Although a minority of clinicians provided ongoing therapeutic engagement through multiple sessions, most appeared to function as a form of ‘triage’, characterized by brief, intermittent support, single-session interventions, crisis management, and frequent referrals to external services. This suggests that school-based clinicians often need to prioritize immediate needs assessment and crisis management rather than ongoing therapeutic treatment and may need a different format of ‘blended care’ (if it is to be useful in schools) than what would be useful in private practice.

##### Ways to use the digital intervention

Clinicians commonly anticipated that the app could be used in-session, with the clinician providing step-by-step guidance and assigning specific activities as homework with in-app reminders. Many suggested it would be most effective for reinforcing concepts already discussed, rather than introducing new material. A few also cautioned that the digital component should not replace in-person teaching of therapeutic skills. However, clinicians frequently noted the digital intervention's value as an in-session reflective tool, emphasizing its usefulness in prompting client recall and insight. In particular, the mood tracker was highlighted as providing concrete evidence of mood between sessions, which is often challenging with younger clients.

Many clinicians also identified the potential broader utility of ClearlyMe® beyond routine therapy, suggesting its potential value during waitlist periods, therapy breaks (e.g., school holidays), and post-discharge transitions. These alternate uses of the app were particularly emphasized by school-based clinicians, in part owing to resource and time constraints, as well as specifically for students receiving single-session or brief interventions.

##### Keeping clients engaged

Most clinicians reported that digital interventions they had previously engaged with are generally easy to use and more contemporary than pen-and-paper approaches for ‘homework’. However, a clear consensus among clinicians was that, regardless of the ease of use, a digital intervention should be used in a way that is not 'too much work' (i.e., not assigning too many activities at once). Building client buy-in and engagement through introductory activities such as the mood tracker, setting achievable goals, giving clients choices and agency, and checking in on digital intervention use were also important to clinicians in ensuring successful and meaningful use of the intervention approach.

##### Flexibility

Given the significant variability in client needs, particularly when working with adolescents, the majority of clinicians were cautious about an overly ‘manualized’ treatment model. Experienced clinicians reflected that earlier in their career, they would have valued a manualised blended care treatment model, but with increased experience, they tend to have a more flexible plan and adapt therapeutic approaches to the current situation.​ One clinician earlier in their career identified that a manual to support their practice would be highly helpful to support their early-stage career anxieties. Clinicians working in schools also noted a misalignment between the practical realities of delivering treatment in school settings and the structured requirements of a manualised approach. It was also very important to clinicians that they not alienate clients with an overly rigid format, particularly those who prefer person-to-person connections over digital tools.

**Table 1:** Subthemes, and illustrative quotes for theme: optimal ways to integrate app with therapy

Sub-theme	Example quote
Introduction to ClearlyMe	[The app] comes out after you've had the long discussion and the rapport building *(12.1S)*
	I love the mood checker component as like a really soft entry *(4.2P)*
	[The app] might come about as a solution to a barrier that we picked up when they didn’t implement the strategies the first time *(18.1S)*
Ways to use the digital intervention	Often, I have to print out like five different skills, because I don't know which one I'm going to end up doing.... So having this [app] there for me would be really helpful *(8.1P)*
	I feel like it’s a real gentle way to maybe assign homework that might not feel like homework *(6.4P)*
Typical pattern/duration of contact	I will quite often just see people as a one-off. That's the thing. They'll drop in. *(12.1S)*
	Six to ten [sessions] for the young people who are coming with one specific goal… the more complex ones are going to be ten, unless their parents can afford to keep paying without a rebate *(6.2P)*
Keeping Clients Engaged	You want to get them to engage, but if it feels like work, they're not going to *(4.2P)*
	Clients use [apps] as long as I keep the motivation up *(9.2P)*
	Having a lot of those homework activities centralised in the app would be really beneficial, because sometimes, you do print something off and you go through it, and then they're like, “I lost it”* (9.5P)*
Flexibility	As a new psychologist, that [manual] was really great to use to sort of plan. But now that I'm an experienced psychologist, I can move around. Like, I don't need a program to be flexible, I just make it flexible *(21.1S)*

#### Suitability of clients for blended care

The next theme identified was varying perceptions regarding client suitability for the blended care model, identifying groups for whom the approach was clearly indicated, potentially contraindicated, or suitable only with adaptations for specific groups.

##### Indicated

The prevailing view among clinicians was that blended care could be appropriate for many clients with low mood. Help-seeking, motivated, and less symptomatic adolescents were identified as ideal candidates for a blended approach. There were differences of opinion regarding clients with comorbid or complex presentations, whereby some clinicians felt it would be beneficial as a supplement to therapy and other support (see subtheme *3.2.2.3. Adaptations for specific groups*), whereas others felt it would not be appropriate (see subtheme *3.2.2.2. Contraindicated*).

##### Contraindicated

A small number of clinicians felt that ClearlyMe would not be suitable for use in blended care for clients with intellectual disabilities, or low literacy, due to the app’s perceived low levels of accessibility for this group (e.g. high levels of text, navigation). Many clinicians were cautious about recommending the app to high-risk clients (or recommending *only* ClearlyMe as a resource), such as those with trauma, suicidality, or self-harm, who may require risk management and safety planning approaches, as these functions are not presently available in the app. Therefore, while blended care may work well for these clients, a program including the ClearlyMe app would require additional risk-based support.

The majority of clinicians confirmed the importance of tailoring the model to individual readiness, personality (for example, highly conscientious clients are more likely to engage), and expectations of therapy. Generally, clinicians viewed clients who exhibited low motivation, ambivalence, or resistance to change as potentially challenging to engage with a blended care model, because of the need for self-directed engagement outside of the face-to-face sessions. Clinicians repeatedly noted that individuals with perfectionistic tendencies may experience heightened pressure from the introduction of a digital intervention, due to their greater desires to ‘get it right’. Additionally, a few clinicians working in schools reflected that the structured nature of blended care might not align with the preferences of students, who are primarily seeking emotional connections or informal support rather than structured therapeutic interventions.

##### Adaptations for specific groups

There were differences in view regarding the app’s suitability for neurodiverse young people, and it should be noted that these are particularly in regard to the current format of ClearlyMe, rather than blended care in general, and were not views brought up by all clinicians. The few clinicians who were supportive felt that the app could be delivered with additional psychoeducation on the concepts in the app, a reward system for engagement, and a greater degree of clinician support. The small number of clinicians who indicated that it would not be suitable felt that neurodiverse young people would need more targeted interventions designed with neurodivergence in mind specifically. Most clinicians felt that clients with neurological conditions which cause intellectual challenges would require modelling and support from carers to engage successfully.

For clients with more complex mental health needs, clinicians indicated that they would use ClearlyMe® in an adapted and flexible way, utilizing it to help with the mood component of their difficulties, but noting that this would likely need to be supported by other apps, such as safety planning apps (Table 2).

**Table 2:** Subthemes, and illustrative quotes for theme: Suitability of clients for blended care

Sub-theme	Example quote	
Indicated		They don’t have to be depressed to use this app. They could just be a bit stuck right now* (6.1P)*
		I think these would be a very motivated student to go and access this, or a very curious student *(10.2S)*
Contraindicated		Young people with any kind of cognitive disabilities and learning difficulties, some of them may struggle *(14.1SW)*
		I'm a little bit hesitant with some of my students, especially when they present as very low and struggling with motivation *(10.1S)*
Adaptions for specific groups		A lot of the time with neurodivergent clients, like, having those examples [in the app activities] almost acts as a bit of like a body double *(6.2P)*
		[clients with additional diagnoses] may find it difficult to understand what to do, how to do or what language to use … I think it would be a benefit having the professional that they’re already working with working through it with them* (6.1P)*

#### Facilitators and barriers to adopting blended care

This theme outlines factors that may serve as facilitators or barriers to the use of the blended care model. The subthemes included barriers and facilitators, which may impact the capacity and willingness of clinicians and clients to use a blended care model. Clinicians described barriers and facilitators in terms of app-person interactions, such as digital literacy, reading level, and individual preferences, rather than inherent features of the app itself. For this reason, the barriers and facilitators are presented in relation to client and clinician factors.

##### Client barriers and facilitators

Many clinicians noted that client motivation could be a barrier to engagement, given that lack of motivation is a core symptom of depression. Some clinicians highlighted that although ClearlyMe generally has broad relevance for adolescents with diverse characters, some cultural identities, such as First Nations Australians, may prefer or respond more positively to an app that has undergone appropriate cultural tailoring. Access to smartphone devices and desire to use an app were also identified as barriers by nearly all participants, though for different reasons, it was reported that some young people are hesitant to overuse their phones (particularly older adolescents), and some may not yet have access to a phone (particularly younger adolescents).

Clinicians expressed that the degree of parent involvement is critical, noting that it could ‘make or break’ the success of the blended care model. Most clinicians emphasized the importance of ensuring that parents remain engaged and supportive without becoming overly intrusive regarding their young person’s use of the app. A few clinicians also strongly felt that the app could provide a ‘shared language’ between clinicians, clients, and parents, which would be very beneficial in cases where the parent is receptive and flexible with the young person’s app use.

##### Clinician barriers and facilitators

A common theme centered on clinician workload; all the clinicians expressed being at (or above) capacity in their workload. Because of this, it was clear that a blended care model should be designed to relieve clinicians of their existing workload and not burden them administratively. The specific requirements for training are discussed in Sect. 3.2.5. Training and support *requirements for those delivering care.* Overall, clinicians indicated that a barrier to use would be their knowledge of and confidence in digital therapy apps as well as their digital competence. A minority of clinicians were also fearful of losing a strong therapeutic relationship with their clients by introducing technology (Table 3).

**Table 3:** Subthemes, and illustrative quotes for theme: Facilitators and barriers to adopting blended care

Sub-theme	Example quote
Clinician barriers and facilitators	I would probably not use it if I didn't feel there was value in it—as well, obviously… if it was like making my job a little harder *(1.1P)*
	That impersonal element of it, I worry about *(10.2S)*
Client barriers and facilitators	I think the biggest barrier would probably be the child's motivation to engage. *(1.1P)*
	It would be nice to assign the app to clients with parents who have good boundaries instead of parents who bulldoze over boundaries *(6.4P)*

#### Data and privacy issues

This theme encapsulated clinician perspectives about the types of app data that would be clinically meaningful, as well as their considerations and concerns regarding client privacy and data security.

##### Data sharing

All clinicians felt that it would be useful to know which content in the app their client had engaged with. However, they largely emphasized that automated data sharing should be minimal and quantitative, with detailed responses shared by the clients at their discretion in sessions. This was driven by a concern that teen clients may feel ‘watched’ and would therefore not authentically engage with the app. Clinicians almost unanimously expressed that the mood tracking feature of the app would be a particularly useful tool for blended care, as many clients have difficulty recalling how they have felt outside of sessions. Some clinicians saw value in a secure web-based portal where clinicians could view client data, although some clinicians preferred to ask the client directly for this information in the sessions. Clinicians reflected that a simple, easy to use, digital thought diary for clients would be a valuable addition to ClearlyMe for use in a blended care model as this aligns with the types of homework activities the participating clinicians were currently assigning to teens.

Finally, clinicians expressed reservations about receiving client information, particularly regarding symptoms or indicators of risk, outside of session hours, highlighting significant concerns related to their duty-of-care obligations and the challenges of adequately responding to risks beyond their capacity.

##### Consent and privacy

Clinicians emphasized the importance of client autonomy, with a few identifying that data sharing needs to be a client-led process with fully informed consent. Clinicians were often concerned that young people may acquiesce to requests for data sharing, feel uncomfortable, or stop using the digital intervention entirely. Clinicians also noted the importance of a clear protocol to terminate data sharing once a client has completed treatment (Table 4).

**Table 4:** Subthemes, and illustrative quotes for theme: Data and privacy issues

Sub-theme	Example quote
Data sharing	The metrics of how often they're using it could be helpful to guide me. Are they going outside of the session and practicing? *(12.3S)*
Consent & privacy	I'm also mindful of their right to privacy and autonomy and being able to kind of do what they want in-between sessions *(14.1SW)*
	Teenagers will say yes to things that they don't want to say yes to in a session … I think there'd possibly be a feeling of an expectation that they have to do that *(4.1P)*

#### Training and support requirements for those delivering care

This theme encompasses clinicians’ preferences for training content, modality of training, and personnel that may be involved in delivery of blended care.

##### Training content

According to clinicians, training should cover the app features, how to navigate the app, which of the app activities map onto specific CBT components, which activities are relevant to which symptoms/presentations, what to set as homework, and security and privacy features​. Some clinicians indicated that case studies or testimonials from young people would be valuable. Most clinicians indicated that they would like a brief, visual guide, using infographics, to explain the model and approach, and a parent information sheet. Clinicians also consistently indicated that they would like to be able to select sections of content (to be highlighted in the app or notified to clients) that can be chosen on the basis of individual client needs and personalized to the client.

##### Training preferences

Private practice clinicians generally agreed that online self-guided or webinar training materials are preferable to in-person training methods. School-based clinicians often endorsed in-person training but emphasized that this should align with existing meetings and professional development opportunities (e.g., fortnightly team meetings in schools and staff development days). Both groups emphasized that time of day for training is important (e.g., before or after school), training should be relatively brief (e.g., a half day is too long) and ideally should have a live presenter who can answer questions​. Across settings, senior staff could be trained and, in turn, could share this with their team. The training could be included as continued professional development.

##### Ongoing support needs

Most clinicians indicated that a monitored email account with high-quality and responsive support would provide sufficient IT support. Quick communication about app updates was also requested. While there was some interest in monthly or drop-in sessions where clinicians can check in and ask questions or discuss issues​, a Community of Practice online forum was a more popular option.

##### Personnel

School-based clinicians highlighted the value of extending the use of ClearlyMe, beyond traditional school psychologists and counsellors to include a broader range of staff who have well-being roles in schools, and who are often the initial contact points for students seeking support. Clinicians noted that digital intervention could significantly increase the capacity of these staff, particularly in contexts where access to formally trained mental health professionals is limited. Additionally, social work practitioners emphasized the importance of explicitly incorporating staff with social work or equivalent backgrounds into intervention models in general, especially in schools facing resource constraints or located in remote areas (Table 5).

**Table 5:** Subthemes, and illustrative quotes for theme: training and support requirements for those delivering care

sub-theme	Example quote
Training content	I would think something that shows you where things are [in the app] would be helpful *(5.2S)*
	I would definitely suggest giving a clinician a letter to send to parents, just in case the child does say, “Yes, you know I—I want my parents to know that I am using this app.” *(19.1S)*
	Which one [app activity] would I give this client? Where would I start? *(3.1P)*
Training preferences	I'd say a combination of both [synchronous and asynchronous training] *(9.4P)*
	[Private practice clinicians] are busy. They're more likely to do an online course *(21.1S)*
	Too much information is not helpful. We don’t have time and it’s too overwhelming *(18.1S)*
Ongoing support needs	Good IT support is needed. Like, oh, the data is gone; what do I do? *(2.1S)*
	It may be helpful to even have that [web portal] as a Community of Practice *(9.1P)*
Personnel	I outreach to rural towns where there are no services to secondary schools…. I think it is very important not to forget the social workers because we provide a lot of that support *(15.1SW)*
	It can’t hurt for them [other wellbeing staff] to say, look, I’ve got this, maybe give it a go; the school counsellor will be in next week *(18.1S)*

## Discussion

The purpose of the current study was to understand clinician preferences to inform the development of a blended care model that integrates a CBT-based smartphone application (ClearlyMe) with face-to-face psychological therapy for adolescents with depression. Overall, clinicians reflected that their use of this type of model would depend on how well ClearlyMe aligned with their existing therapeutic approaches. Rather than replacing core elements of therapy or reducing their professional input, clinicians perceived ClearlyMe to be most useful as a complement to their practices for enhanced treatment continuity outside of sessions. Clinicians also reflected that their professional perceptions of client suitability would shape their decisions to adopt this model.

While ClearlyMe was considered suitable for many adolescent clients experiencing depression, clinicians perceived its greatest utility in cases where adolescents were highly motivated, noting that engagement strategies may need to be adapted for those with more severe or complex presentations. This aligns with previous research from adult blended care models, which has shown that digital components are often more effective when users are engaged and motivated [28]. However, in contrast to structured adult models aimed at streamlining care or reducing clinician workload, clinicians in this study did not view ClearlyMe as a tool to increase treatment efficiency or replace sessions. Instead, they saw it as a flexible way to extend therapeutic engagement beyond the sessions and between sessions, or to provide psychoeducational support and "homework" outside of therapy. This is a particularly important finding as it indicates that a highly structured, manualized blended care model may not meet the needs of clinicians working with adolescents. Rather, clinicians emphasized the importance of flexibility and clinical autonomy, specifically, the ability to determine who should use the app, when, and in what capacity. Blended models for youth must be tailored not only to developmental needs but also to the structural realities of service provision, particularly in highly variable educational settings which are not relevant to adult models.

Additionally, clinicians consistently highlighted the mood tracking feature as broadly applicable and valuable across diverse youth populations, noting that simple mood tracking tools are often difficult to find. They viewed this feature as an accessible entry point to engage adolescents, a useful in-session reflective tool, and a way to provide concrete evidence of mood between sessions. While other features were considered helpful in specific contexts, mood tracking emerged as the most universally endorsed component.

Regarding practical considerations, clinicians expressed that data handling and privacy conditions within the app would influence their decision to adopt a blended care model. This is consistent with the themes raised in the co-design of the app initially, with mixed views on the capabilities for data sharing [14]. Clinicians also raised concerns about clients’ comfort with the sharing of their personal information through a digital platform, which clinicians feared may impact how fully a young person would engage with the platform if they felt ‘watched’. Training in the model was viewed as critical to successful implementation, with clinicians preferring brief, remotely delivered training with examples relevant to various professional contexts. Flexibility in treatment delivery, such as asynchronous or modular formats (both in terms of training, and modules within the digital tool), was considered essential to support uptake, particularly given the variability in session availability and clinicians’ capacity to offer consistent, ongoing care across different service settings. 

Previous research into blended care [8] indicates that while clinicians generally have positive perceptions, practical barriers related to workflow, client characteristics, and system-level support persist, echoing themes raised in this study. These implementation challenges are particularly important given the consistent findings of systematic reviews (e.g., [26]), which show that digital interventions are most effective when paired with human support, but also shows the importance of tailoring these models to a youth audience. This highlights the clinical value of blended models, which preserve therapeutic contact while extending reach through digital means [13]. A recurring issue in the literature is low adherence to self-guided digital programs,while in contrast, meta-analyses have shown that clinician-supported interventions achieve better engagement and outcomes in adults (e.g., [15]). Whether this is also the case for adolescents remains an important area for future investigation, cementing the value of additional adolescent-tailored research.

### Implications for the design of a blended care model integrating ClearlyMe

The findings of this study highlight several considerations critical for users of digital mental health tools in a blended model. First, this research identified unique barriers in school settings. While school-based clinicians were generally supportive of using tools such as apps to support their practice, the heterogeneity of school-based practice does not support a multisession, structured (or semi structured) manualized approach. Owing to time constraints, one-off (‘drop-in’) sessions, and limited continuity of care, a single-session model or brief materials provided to educate school staff about digital resources would be more appropriate in this context.

Second, tools must address a range of clinician concerns to ensure relevance and encourage adoption. Without clear and demonstrable utility, their adoption and sustained use are unlikely. Clinicians are often time-poor and face significant administrative burdens, and any blended care model that adds to this workload (including onerous training requirements) is unlikely to be widely adopted by clinicians. A blended care model should make a clinician’s job more efficient, such as providing resources that clinicians already assign in other formats (such as print-out activities) in one straightforward, easy-to-use platform.

These findings contribute to the limited body on clinician perspectives toward blended care for adolescents, particularly within the Australian context. Notably, Australia’s health provision infrastructures, including Medicare-subsidized sessions facilitate integration. However, translating these findings to other countries will require careful consideration of differing healthcare funding models, service delivery systems, and sociocultural factors. The international applicability of these findings should therefore be evaluated carefully.

To summarise, this research has identified that blended care models targeted towards young people should be brief, flexible, and easily integrated into diverse practices, emphasizing modularised formats that align with the realities of clinician’s busy and full workloads. A successful model would ideally minimize administrative burden, demonstrate clear utility, and offer streamlined access to commonly used resources within a user-friendly platform. This includes adaption of existing ‘paper-and-pen’ activities that clinicians may use to a digital format, and utilisation of mood tracking measures to promote engagement and self-reflection. To ensure relevance and adoption, the model should support rather than complicate clinician workflows. Training formats should be flexible, with online modules preferred for those who work in private practice, and face-to-face or group online sessions preferred for a model designed for schools. Finally, while a multi-session modularised model would suit private practice, school-based blended care needs to account for one-off, brief interactions, with limited time and resources.

## Limitations

While the sample had strong representation from private practice and school-based clinicians, different views may be found among those working in public health settings or more structured service models (e.g. headspace). Clinicians in rural or remote regions may also face different access challenges and have unique perspectives on the feasibility of blended models. While some clinicians in the current study reported working in regional and remote areas during the interviews, this was not something that was measured in the current study. This limits the degree to which we can comment on the applicability of the findings to diverse groups working in different areas and will need to be assessed in future studies.

An important limitation is that clinicians’ perspectives were necessarily speculative, as they were, for the most part, based on a brief trial period with the app rather than extended real-world use. While some clinicians reported using the app for extended periods of time, or indeed already using the app with clients, this was not formalized and varied significantly amongst the group. Their views therefore largely reflect anticipated feasibility and challenges, which may differ from experiences in practice.

Finally, although participants were encouraged to be critical in their feedback, and interviewers emphasised the value of critical perspectives, it is possible that some participants may have moderated their responses given the interviewers’ role in the research team to give socially desirable answers. As such, there remains a possibility that participants were less candid in their discussions than they otherwise would have been with an interviewer who was explicitly neutral.

## Conclusions

This study highlights both the potential and challenges of blended care approaches to mental health treatment for adolescent depression. While clinicians were generally receptive to using digital tools like ClearlyMe® within a blended care model, their uptake is contingent on alignment with existing workflows, perceived clinical utility, and flexible implementation options. The development of flexible, problem-solving digital tools, paired with ongoing clinician support and alignment with systemic structures appears to be the most promising path forward. Continued research and codesigned innovations will be crucial to optimizing the role of blended care in future mental health service delivery, to ensure that blended care models are not only effective but also feasible, acceptable, and scalable across diverse service settings.

## Key points and relevance

- The rates of youth depression are increasing, yet access to timely care remains limited. Blended models may help overcome these barriers by integrating digital tools with face-to-face support.
- The present study explores mental health professionals’ perceptions of the role that a CBT-based app can play in their existing practices with adolescents.
- Clinicians indicated that blended care models should build on or streamline resources they already use, for example automating homework tasks.
- Key concerns include low client engagement, the privacy of client information, and digital literacy barriers for both clinicians and clients.
- Successful implementation will require organisational support and concise, flexible training that preserves clinician autonomy within the model.

## Supplementary Information

Additional file 1.

## Data Availability

The datasets generated and analysed during the current study are not publicly available due to the sensitive and personal nature of qualitative data transcripts. In accordance with participant consent, data may only be used for the purposes of this research study. Coded data may be made available by the corresponding author upon reasonable request, subject to conditions that ensure compliance with the original scope of participant consent and ethical requirements.
