# Supplementary material for: Clinician perspectives about delivering a blended care treatment model for adolescent depression: a qualitative study
Source: Child Adolesc Psychiatry Ment Health. 2025 Nov 21;19:132. doi: 10.1186/s13034-025-00989-z (PMC12639716; doi:10.1186/s13034-025-00989-z)
Supplement: Supplementary file 1 — Additional file 1. [file 13034_2025_989_MOESM1_ESM.docx]

Supplementary Material 1:

*Focus group for psychologists*

1. **Introduction (5 mins)**
2. **Confidentiality (1 min)**

Before we start, we would just like to remind everyone about the importance of maintaining confidentiality. Although we will likely not be discussing personal or sensitive matters, we ask that you do not share any information from the focus group with any other party.

1. **Power point introduction app (5 mins)**
2. **Time allocation to use the app (10 mins)**
3. **Focus group questions (approximately 60 minutes)**
4. How should the ClearlyMe® digital intervention be integrated with the ways psychologists currently deliver person-to-person therapy?
5. What features/symptoms would make a client suited or not suited to blended care treatment?
6. How prescriptive or flexible should the treatment model be?
7. What features of the blended care model are required in different settings (e.g., private practice vs schools)?
8. What should the blended model look like in terms of length and frequency of treatment? How often would you recommend that your clients engage with the app vs engage with the treating professional?
9. What are your views about a fully manualised treatment programme delivering CBT for mild–moderate depression (8--10 sessions), which specifies how to integrate and best personalize ClearlyMe® for your client?
10. What information/data would you want to know from the app while your client is using it?
11. What are the training requirements for professionals to implement this model?
12. What support do professionals require while implementing the treatment model?
13. What (if any) involvement may parents want?
14. What are the barriers to the implementation and use of this model?
15. What are your suggestions about how these barriers can be overcome?
16. What would make it easier for you to integrate this into your current practice?
17. What are some of the reasons you might not use a blended model like this?
18. What haven’t I asked that I need to know about something like this?
19. **Closing and next steps**
